# *Brachiaria ruziziensis* Responses to Different Fertilization Doses and to the Attack of *Mahanarva spectabilis* (Hemiptera: Cercopidae) Nymphs and Adults

**DOI:** 10.1155/2014/543813

**Published:** 2014-01-22

**Authors:** Daniela de Melo Aguiar, Alexander Machado Auad, Marcy das Graças Fonseca, Melissa Vieira Leite

**Affiliations:** Embrapa Dairy Cattle Research Center, Rua Eugênio do Nascimento, 610 Dom Bosco, 36038-330, Juiz de Fora, MG, Brazil

## Abstract

Cropping practices are necessary in order to help reduce the population of pest insect, such as the induction of resistance through fertilization. Therefore, this study aimed to assess alterations on the production and quality of *Brachiaria ruziziensis* when receiving the fertilization composed by the macronutrients NPK and/or exposed to the attack of *Mahanarva spectabilis* nymphs and adults. *B. ruziziensis* plants were fertilized according to the recommendation (R), half of the recommended fertilization (H), or non-fertilization (C). They were also exposed to different *M. spectabilis* nymph and adult densities. The damage, regrowth, and bromatological components were evaluated. The fertilization treatment promoted a higher *M. spectabilis* nymph survival on *B. ruziziensis*; however, it reduced the damage caused by the forage exposed to nymphs and adults of pest insect, and it did not alter the quality of the signal grass. Moreover, the fertilization treatment enabled forage recovery, even when exposed to 5 nymphs or 10 spittlebug adults.

## 1. Introduction

The livestock is one of the most important Brazilian economic activities, and its contribution for the gross domestic product (GDP), in the second semester of 2011, achieved 61.9 billion reais [1]. Among the forages used in Brazil, it is possible to point out *Brachiaria ruziziensis*, because it contains a high quality for the cattle. Nevertheless, productivity levels in most Brazilian pastures are considered low, due to their degradation level [2].

Besides, forage productivity is also compromised by the spittlebugs attack [3]. This fact is worrying, because more than 90% of cattle for meat production use forages for feeding [4], as they represent one of the most economic food resource [5]. It is not known if cercopids affect the chemical-bromatological composition of forages. This is represented by crude protein content, acid detergent fiber, neutral detergent fiber, and values of *in vitro* dry matter digestibility that are important on the quantitative analysis of the forage, as long as these variables might assume direct or indirect influence on the consumption of dry material and, consequently, on the animal production [6].

Among the species that occur in Brazil, *Mahanarva spectabilis* (Distant, 1909) is considered a limiting pest upon the forage grass production [7, 8]. These insects, nymphs and adults, cause harm to the host plant [9] by sucking its sap and injecting a toxin, that initiates yellowing and drought of the forage [10]. This makes it unpalatable for the cattle [11]. The damage caused by the spittlebugs is around 840 to 2100 billion dollars per year throughout the world [12], justifying the necessity of discovering efficient control methods for minimizing these losses.

There are controlling techniques, and the one which has the most succeeded is the diversification of pastures by using resistant grass [13–15]. The control by forage resistance has low cost and may be easily adopted by the agricultures; however, getting resistant materials demands a long wait. The adoption of chemical control is not recommended, because it is economic and ecologically unfavorable [16]. Therefore, it is strongly necessary to develop good cropping practices in order to help reduce the population of this pest insect, also aiming to answer the producers demand, such as the induction of resistance through fertilization.

Morphological responses from plants to fertilizers are evident, such as changes on growing rates, acceleration or delay of maturity, size of parts of the plants, and the thickness and hardness of epicuticle, which influence the success of many pest species when attacking the host [17]. Moreover, any factor that affects the plant physiology may lead to changes on the resistance to pest insects [17].

Thus, it was aimed to verify changes on *B. ruziziensis *production and quality, when fertilized with macronutrients NPK and/or exposed to *M. spectabilis *nymph and adults attack through evaluation of damage score, regrowth, and bromatological components.

## 2. Material and Methods

### 2.1. *Brachiaria ruziziensis* Plants and Pest Insect Maintenance

Seeds of a commercial variety of *B. ruziziensis* were planted in trays containing vermiculite and after 20 days were transferred to plastic tubes (35 cm^3^) containing plant substrate. After 35 days, two seedlings were moved into vases with 1 Kg capacity of soil collected from the field with clay texture (59% of clay, 5% of silt, and 36% of sand). In order to correct the soil's acidity, dolomitic calcarium (PRNT 90%) was applied. The plants were kept in a greenhouse (average temperature 27.5 ± 4°C).

*M. spectabilis *nymphs and adults were collected in Embrapa Dairy Cattle Research Center, Brazil, and kept in the entomological laboratory and greenhouse. Recently emerged adults were transferred to acrylic cages (30 × 30 × 60 cm). The cages contained a section with gauze moistened with distilled water, which served as an oviposition substrate. To remove the eggs deposited on the substrate, the gauze was placed on a set of sieves and subjected to water jets, such that the eggs remained on the thinnest sieve (400 *μ*m mesh size). The eggs were placed in petri dishes (2 cm high × 5 cm in diameter) lined with moistened filter paper and kept in a climatic chamber at 25 ± 2°C, with 70 ± 10% relative humidity (RH) and a 14 h photophase. The filter paper was moistened daily, and embryonic development was observed up to stage 4 (S4), close to hatching, which was used on the experiments. This developmental stage of the egg is characterized by 2 red spots on each side of the operculum, corresponding to the eyes, with the red spot representing the nymphs' abdominal pigments [18].

### 2.2. Plant Fertilization Effect on the *M. spectabilis *Nymph Survival

Plants roots were exposed and filter paper sections (1 × 2 cm), with sixteen eggs (S4) of *M. spectabilis*, was placed in each vase.

After the soil analysis, the plants were fertilized according to the literature recommendation (R) [19]. This fertilization was done applying 45 mg/dm^3^ of urea, 255 mg/dm^3^ of superphosphate, and 28 mg/dm^3^ of potassium chlorate on the planting and 140 mg/dm^3^ of NPK 20-5-20 on the 30th and the 60th days. The same number of plants received half of the recommended fertilization (H). The control group (C) received no fertilization.

The vase and the signal grass base were kept in fabric bags (voil), tied to the plant base, to prevent the insects from escaping. After 32 days, the nymph survival percentage was recorded on the plants submitted to fertilization doses R, H, and C. The assays were conducted in a randomized block, with three treatments containing 40 repetitions each, totalizing 120 experimental units. Data were submitted to variance analysis and the averages were compared by the Tukey test (*P* < 0.05), using the Sisvar program [20].

### 2.3. Plant Fertilization Effect and/or *M. spectabilis* Nymphs Attack on the Damage Score, Regrowth, and Bromatological Components

Forty days after pruning, *B. ruziziensis* was fertilized as described for experiment one. Plants submitted to fertilizing doses R, H, and C received 0, 5, 15, or 30 nymphs of 4th and 5th instars. For 10 days, plants were evaluated daily and the dead nymphs and the emerged adults were taken out. At the same time, nymphs were restored in to order to maintain the density of each treatment. After this period, nymphs were taken out, and for each plant the damage scores were given, which were adapted to Cardona et al. [21] scale, by three evaluators; moreover, the plants height was assessed. In the next step, the shoots of the plants submitted to the fertilizing dose R, H, and C infested with 30 nymphs or in the absence of them, were cut 7.5 cm above the soil and weighed, dried in an oven at 55°C, and grinded for the chemical-bromatological analysis: lignin, cellulose, *in vitro* dry matter digestibility (IVDMD), neutral detergent fiber (NDF), acid detergent fiber (ADF), and crude protein (CP) using nIR (near infrared reflectance). The regrowth was assessed 30 days after being cut.

The assays were conducted in a randomized block, with four insect densities (0, 5, 15, and 30) and three fertilization doses (recommended, half of recommended, and control doses), with ten repetitions, totalizing 120 experimental units.

To evaluate the fertilization effect on the damage score and the regrowth, the data were submitted to variance analysis and the averages were compared by the Tukey test (*P* < 0.05), whereas, to evaluate the nymph density effect, data were submitted to regression analysis. For bromatological quality of *B. ruziziensis *the assays were conducted in a randomized block design in a factorial arrangement with two densities (0 and 30) and three fertilization doses (R, H, and C). The data were submitted to variance analysis and the averages were compared by the Tukey test (*P* < 0.05), by the Sisvar program [20].

### 2.4. Plant Fertilization Effect and/or *M. spectabilis* Adults Attack on the Damage Score, Regrowth, and Bromatological Components

*B. ruziziensis* plants were submitted to different levels of fertilization, as described in experiment one. Sixty days after the second maintenance fertilization, each vase containing signal grass was put inside a metal cage (70 × 40 × 40) covered with organza fabric. Ten *M. spectabilis* adults (five males and five females) were released into each cage or the adults were absent.

Daily, the dead adults were taken out and the same quantity of males and females was restored in order to keep the density of each treatment as well as the male/female ratio. After five days, spittlebugs were taken out and three evaluators attributed the damage score for each plant. Plants were assessed for five days, a period sufficient to visualize the damage, based on previous tests.

The shoots were cut 7.5 cm above the soil and dried in an oven with air circulation for seven days at 55°C. After being dried, the plants were grinded in order to analyze lignin, cellulose, *in vitro* dry matter digestibility (IVDMD), neutral detergent fiber (NDF), acid detergent fiber (ADF), and crude protein, using the device nIR (near-infrared reflectance). The regrowth was evaluated 30 days after being cut.

The assays were conducted in a randomized block design in a factorial arrangement 2 × 3, with two insect densities (0 or 10) and three fertilizations (recommended, half of recommended, and control ones), with seven repetitions, totalizing 42 experimental units.

To evaluate the fertilization effect and the adult's density on the damage score, regrowth, and bromatological component, data were submitted to variance analysis and the averages were compared by the Tukey test (*P* < 0.05), by the program of Sisvar [20].

## 3. Results and Discussion

### 3.1. Plant Fertilization Effect on the *M. spectabilis *Nymph Survival

**Figure 1:**
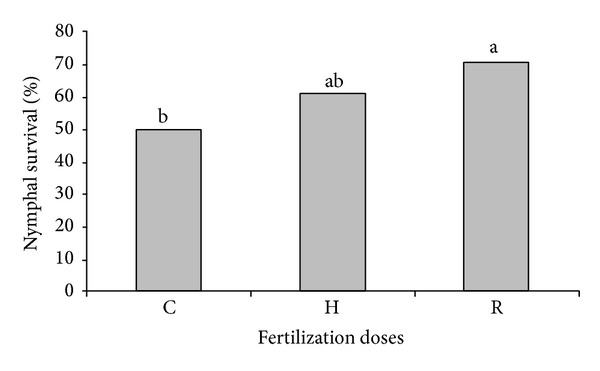
*Mahanarva spectabilis *nymph survival (%) kept in *B. ruziziensis* cultivated under recommended (R), half of recommended (H), and control (C) fertilization. Means values followed by the same letter did not differ among them by the Tukey test (*P* < 0.05).

Fertilization promoted a significant difference on the *M. spectabilis* nymph survival (*F* = 5.448, *P* = 0.0062), being 1.4 times higher when nymphs were kept in plants which received the recommended fertilization dose compared to the control group. The nymphs kept in plants which received half of recommended fertilizer dose were similar to those kept in plants which received no fertilizer and to the ones which received the ideal fertilizer dose (Figure 1). It is suggested that the higher survival will cause population increase of the pest insect in pastures fertilized with NPK, in agreement with Butler et al. [22] who reported a significant effect on the insect population, when NPK elements were used together.

Management of soil fertility might assume several effects upon the plant quality, which might affect the insect abundance. Some studies documented a higher survival of certain insects when plants received fertilization [23–25]. Nevertheless, how the fertilizers act on the insect population is an area of intense debate, sometimes even contradictory, as a few research results recorded positive, negative, or neutral effects of the fertilizers on the insect population [22].

The crop fertilization might affect plant susceptibility to pest insects by affecting plant resistance to attack or by altering plant acceptability by certain herbivores [17]. Regardless of fertilization, *M. spectabilis* nymph survival changed from 50 to 70% and, according to Cardona et al. [21], a genotype is only considered resistant to spittlebugs when the survival is lower than 30%, confirming the *B. ruziziensis* susceptibility to *M. spectabilis* nymph survival. Treatment of plants with NPK recommended fertilization implies that this cropping practice is not sufficient to make *B. ruziziensis* change its status from susceptible to resistant to the cercopid.

Although fertilizer application is important for the crop yield, it is necessary to determine the cost benefit of the relation between the maximum crop yield and the susceptibility to pest insect attack on the fertilized crop [26]. Therefore, this crop method should be used with restriction in areas where *M. spectabilis *reveals pest troubles, since survival levels were high in plants fertilized with NPK. However, the NPK fertilization effect on plant tolerance to the cercopid nymphs and adults attack also should be taken into consideration.

### 3.2. Fertilization Effect and/or *M. spectabilis* Nymph and Adults Density on *B. ruziziensis* Tolerance by the Damage Score

**Figure 2:**
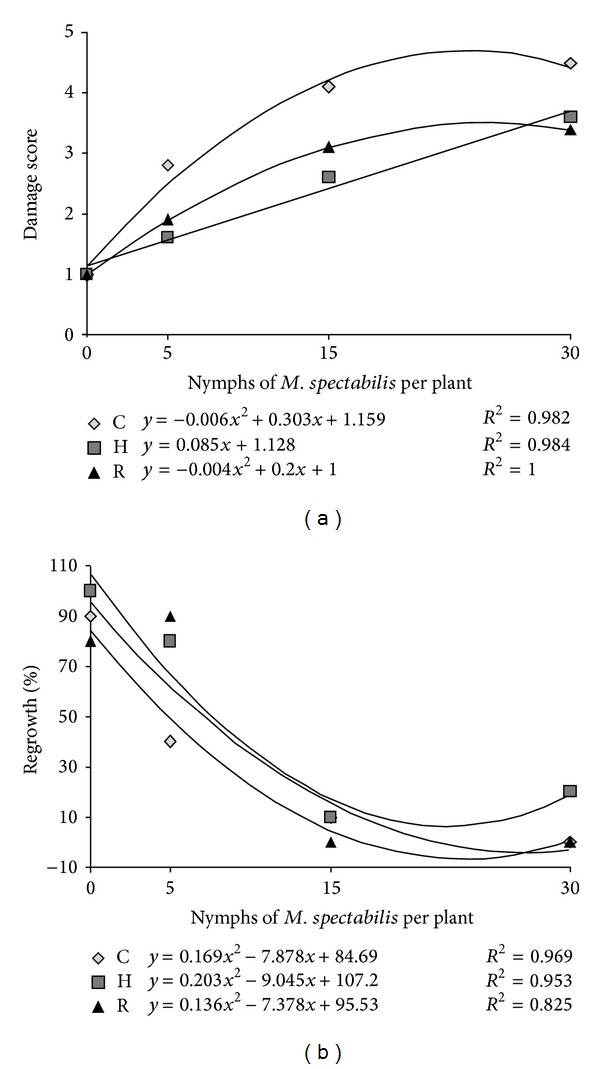
Leaf damage (a) (damage score) and regrowth (%) (b) of *Brachiaria ruziziensis *cultivated under recommended (R), half of recommended (H), and control (C) fertilization and exposed to different *Mahanarva spectabilis* nymphs densities.

The damage of the leaf area increased significantly in nonfertilized plants (*P* < 0.001 and *F* = 79.107) and on those that received ideal fertilization (*P* < 0.001 and *F* = 40.094) with increasing nymph density from 0 to 15 nymphs and leveling off when plants were maintained with 30 nymphs. The data formed quadratic regressive curves with high correlation coefficients (Figure 2(a)). For the plants that received half of recommended fertilization (H), a significant increase in the damage of leaf area was observed (*P* < 0.001 and *F* = 43.713) with reference to pest insect nymph density, forming a linear model (Figure 2(a)). Plants fertilized and attacked by five, fifteen, and thirty *M. spectabilis* nymphs displayed a significant reduction on the leaf damage, compared to those kept in soils without fertilization (Table 1).

Ideal fertilization provides a suitable balance among the essential elements to the plant, which might stimulate the resistance to the insect attack [27], through secondary compounds that can modify the food quality and reduce palatability [28]. This was confirmed in the present research, where the three essential elements NPK used together promoted tolerance for *M. spectabilis* nymphs, confirming results of Fernandes et al. [29] that recorded *Coffea arabica* tolerance fertilized with high N and K concentration to *Coccus viridis* attack. According to Miyasaka et al. [30], the use of N and K isolated or together is fundamental in the insect response to the plant attack, whereas N or NK promoted increase in the aphid *Sipha flava* response to the *Pennisetum clandestinum* attack; however, no difference was observed on the injuries of these aphids when only potassium was applied.

Nitrogen is a limiting component of the homopteran diet [31] and these insects might consume daily up to 100 times its own body weight to obtain enough nitrogen [30], as this element is crucial for the insect's survival [29, 32]. We suggest that nitrogen existing in the fertilizers used on *B. ruziziensis* plants may have reduced *M. spectabilis *herbivory, as low consumption was sufficient for obtaining their necessary nitrogen. In addition, the potassium builds resistance in the cellular walls [33], which might have promoted the reduction of spittlebugs feeding rate, consequently, reducing damage of the plants.

Damage of leaf area increased significantly in nonfertilized plants and in those which received the half of recommended fertilization and recommended fertilization, when exposed to 10 adults, compared to plants exposed to zero *M. spectabilis* adults (Table 1), indicating that this insect density is enough to cause damage regardless of the applied fertilization. Nevertheless, within the density of 10 adults, injuries on the nonfertilized plants were observed (control group) in more than 68% of the leaf area (3.9 on the damage score), reducing significantly the damage verified on fertilized plants with the recommended dose, or half of this one. These results confirm those from Scanavachi et al. [34] and De Bortoli et al. [35], who observed higher damage when there was no fertilization in corn and sorgo, exposed to its main pests. In this study, when the plants were fertilized, the damage averages were not higher than 50% (2.7 on the damage score) (Table 1). Higher injuries (75%) on *B. ruziziensis* fertilized with 50 kg·ha^−1^of NPK exposed for 10 days to nine spittlebugs *Aeneolamia varia *(Fabricius) were recorded by Cardona et al. [21].

### 3.3. Fertilization Effect and *M. spectabilis* Nymphs and Adults Density on *B. ruziziensis* Tolerance by Regrowth

It was verified that a *M. spectabilis *nymph density increase caused reductions in regrowth percentage for nonfertilized plants (*P* < 0.001 and *F* = 15.093), with half of fertilization (*P* < 0.001 and *F* = 18.573) as well as for those which received the recommended fertilization (*P* < 0.001 and *F* = 22.998) (Figure 2(b)), forming quadratic regressive curves with high correlation coefficients. It is suggested that this might have occurred due to the great amount of sap sucked out by the nymphs, impoverishing the plant from its reserves and compromising its regrowth capacity. According to Valério [36], the frequent attacks from *Mahanarva *sp. might reduce the volume of radicular system and decrease grass persistence.

When nymphs are absent, the regrowth was significantly equal, regardless of the fertilization. In low density (5 nymphs), it was observed that fertilization promoted higher regrowth percentage. In this way, Valério [36] emphasizes that well-managed pastures and fertilized soils are less vulnerable to the pest attack. However, in higher densities (15 and 30) there was no effect of fertilization on the regrowth, which did not reach 20% (Table 1). Considering the low percentage of regrowth of the plants submitted to 15 *M. spectabilis* nymphs, regardless of the fertilization, it is recommended that forage by grazing animals, or even cutting and storage when the density of insect per plant is lower than 15 nymphs; it guarantees the persistence of the forage. Moreover, fertilization is only recommended in pastures with low nymph density, which according to Auad et al. [37] normally occurs at the beginning of the rainy season.

When *B. ruziziensis* was exposed to 10 *M. spectabilis* adults, the regrowth was significantly reduced in nonfertilized plants and in those which received the half of fertilization. However, this alteration was not seen in plants which received the recommended dose of fertilization (Table 1). There was 100% regrowth in plants that were not exposed to adults, regardless of the application and dosage of fertilizer, which was different from the Cecato et al.'s [38] results, who observed a regrowth increase in *B. brizantha*, marandu cultivar, with the application of increasing N and P doses.

Nevertheless, when the plants were exposed to 10 adults, regrowth did not occur in signal grass nonfertilized plants, differing from those which received half of recommended dose and the recommended dose, treatments whose regrowth rates were 71% and 86%, respectively (Table 1). The relation between the damage caused by 10 adults and regrowth was evident. Nonfertilized plants showed a 3.9 damage score, and they did not have regrowth while those which were fertilized, having a damage score of at least 2.7, had regrowth over 70%. This result confirms that the deleterious effect of the pest was mitigated, causing tolerance promotion when the macronutrients NPK were supplied. Fertilizers might promote pasture recovery when *M. spectabilis* pressure is high. Meyer [39] argues that the nutrient availability in the soil affects damage caused by herbivores and the capacity of plants to recover. Nevertheless, both factors are rarely taken into consideration together.

### 3.4. Effect of Fertilization and *M. spectabilis* Nymphs and Adults Density in the Quality of *B. ruziziensis*

Fertilization did not promote significant effects on the cellulose, lignin, ADF, and NDF contents and, consequently, on the *B. ruziziensis* IVDMD when *M. spectabilis* nymphs and adults were absent (Table 2). Rosa et al. [40] did not check for a nitrogen fertilization effect on the *B. decumbens *cellulose content and IVDMD; however, they recorded reduction in lignin content. In the same way, Cecato et al. [38] did not verify an effect on *B. brizantha*, marandu cultivar, digestibility submitted to nitrogen fertilization.

When the plants were exposed to 30 nymphs or ten adults, fertilization did not promote a significant difference on cellulose percentage, NDF, ADF, and IVDMD. Lignin was not altered in plants submitted to the attack of ten adults, regardless of the fertilization. However, there was a significant lignin content increase in the recommended fertilization plants in the presence of 30 nymphs of the pest insect but this alteration was not sufficient to change IVDMD.

There was a significant cellulose increase in nonfertilized plants and NDF in those with the recommended fertilization and exposed to 30 cercopid nymphs, compared to those without the insect presence. This difference was not observed for lignin, ADF, and IVDMD. Moreover, in fertilized plants exposed to 30 nymphs there was no significant alteration in cellulose, lignin, ADF, NDF, and IVDMD contents compared to those in which nymphs were absent (Table 2).

We observed a significant decrease in cellulose content only with the recommended fertilization in plants exposed to 10 adults, compared to those without bugs. The same was not verified for plants without fertilization and with half fertilization, regardless of the adult density. Lignin, ADF, and NDF content were not altered by the adult density, regardless of the fertilization (Table 2).

When the plants were submitted to different fertilization doses and *M. spectabilis *nymph and adult density, there were no significant effects on the crude protein content percentage of *B. ruziziensis* (Table 2). It is worthy emphasizing that the present study crude protein values were lower than the critical value of 7% [6], a limiting condition to animal production. Considering that the crude protein content is directly related to the forage age, it is suggested that the results found occurred due to forage growth, as a result of 52 days after being cut, because tropical grasses have their protein and fiber content reduced as they grow older, which results in a low digestibility and nutritional value of the forage [41].

The assessment of *M. spectabilis* effect on *B*. *ruziziensis* quality is unpublished. We highlighted that 30 nymphs, or 10 adults, attack of this pest insect did not promote alteration on forage quality.

Fertilization with NPK promoted greater nymph *M. spectabilis* survival on *B. ruziziensis*; however, it reduced damage by nymphs and adults of the pest insect and did not alter the forage quality. In addition, fertilization was sufficient for the forage recovery, when exposed to 5 nymphs or 10 adults of *M. spectabilis*.

## Figures and Tables

**Table 1:** Leaf damage (damage score) and regrowth (%) of *Brachiaria ruziziensis* cultivated under recommended (R), half of recommended (H), and control (C) fertilization and exposed to different *Mahanarva spectabilis* nymphs and adults densities.

Fertilization doses	Nymphs density			
	0	5	15	30
Leaf damage				
C	1.0 ± 0.0 A	2.8 ± 0.3 A	4.1 ± 0.3 A	4.5 ± 0.2 A
H	1.0 ± 0.0 A	1.6 ± 0.1 B	2.6 ± 0.2 B	3.6 ± 0.3 B
R	1.0 ± 0.0 A	1.9 ± 0.2 B	3.1 ± 0.2 B	3.4 ± 0.1 B

*P *	*1.000 *	* <0.001 *	* <0.001 *	*<0.001 *
*F *	*0.000 *	*12.490 *	*17.709 *	*10.668 *

Regrowth (%)				
C	90.0 ± 10.0 A	40.0 ± 16.3 B	10.0 ± 10.0 A	0 ± 0.0 A
H	100.0 ± 0.0 A	80.0 ± 13.3 A	10.0 ± 10.0 A	20 ± 13.3 A
R	80.0 ± 13.3 A	90.0 ± 10.0 A	0.0 ± 0.0 A	0 ± 0.0 A

*P *	*0.3871 *	*0.002 *	*0.728 *	*0.289 *
*F *	*0.948 *	*6.639 *	*0.316 *	*1.243 *

Fertilization doses	Adults density			
	0	10	*P *	*F *

Leaf damage				
C	0 ± 0.0 Ab	3.9 ± 0.14 Aa	*<0.001 *	333.333
H	0 ± 0.0 Ab	3.1 ± 0.14 Ba	*<0.001 *	187.500
R	0 ± 0.0 Ab	2.7 ± 0.18 Ca	*<0.001 *	120.000

*P *	*1.000 *	*<0.001 *		
*F *	*0.000 *	*27.220 *		

Regrowth (%)				
C	100.0 ± 0.0 Aa	0.0 ± 0.0 Bb	*<0.001 *	52.500
H	100.0 ± 0.0 Aa	71.4 ± 18.4 Ab	0.047	4.286
R	100.0 ± 0.0 Aa	86.7 ± 14.3 Aa	0.309	1.071

*P *	*1.000 *	*<0.001 *		
*F *	*0.000 *	*22.143 *		

Means values followed by the same small letter in the row and by the same capital letter in the column are not significant (ANOVA followed by a Tukey test (*P* ≤ 0.05)).

*P* and *F* are values of variance analysis.

**Table 2:** Cellulose, lignin, neutral detergent fiber (NDF), acid detergent fiber (ADF), *in vitro* dry matter digestibility (IVDMD), and crude protein (CP) contents in *Brachiaria  ruziziensis* plants, cultivated under recommended (R), half of recommended (H), and control (C) fertilization and exposed to different *Mahanarva  spectabilis* densities.

Fertilization doses	Nymphs density		*P *	*F *	Adults density		*P *	*F *
	0	30			0	10		
Cellulose (%)								
C	40.0 ± 2.2	49.6 ± 2.3	*0.015 *	*6.352 *	43.4 ± 3.1	47.7 ± 1.2	*0.206 *	*1.674 *
H	45.5 ± 3.4	49.3 ± 3.5	*0.304 *	*1.083 *	44.1 ± 2.6	44.7 ± 1.3	*0.785 *	*0.076 *
R	45.6 ± 1.8	46.4 ± 1.6	*0.818 *	*0.054 *	47.6 ± 2.6	41.9 ± 2.7	*0.035 *	*4.88 *

*P *	*0.233 *	*0.648 *			*0.320 *	*0.095 *		
*F *	*1.448 *	*0.434 *			*1.171 *	*2.526 *		

Lignin (%)								
C	5.7 ± 0.3	5.5 ± 0.2	*0.508 *	*0.445 *	6.3 ± 0.3	6.1 ± 0.2	*0.415 *	*0.683 *
H	5.8 ± 0.2	5.7 ± 0.3	*0.700 *	*0.15 *	6.3 ± 0.1	6.0 ± 0.2	*0.309 *	*1.072 *
R	6.1 ± 0.3	6.3 ± 0.2	*0.579 *	*0.311 *	6.5 ± 0.3	6.5 ± 0.3	*0.983 *	*0.000 *

*P *	*0.285 *	*0.023 *			*0.601 *	*0.118 *		
*F *	*1.277 *	*4.064 *			*0.512 *	*2.272 *		

DNF (%)								
C	71.2 ± 0.9	73.6 ± 0.9	*0.070 *	*3.447 *	77.0 ± 0.7	75.9 ± 0.4	*0.15 *	*2.172 *
H	73.7 ± 0.9	75.8 ± 1.1	*0.111 *	*2.641 *	75.9 ± 0.5	75.0 ± 0.4	*0.264 *	*1.292 *
R	72.4 ± 1.0	75.8 ± 0.9	*0.015 *	*6.356 *	76.2 ± 1.0	75.9 ± 0.5	*0.721 *	*0.130 *

*P *	*0.152 *	*0.205 *			*0.32 *	*0.401 *	
*F *	*1.949 *	*1.624 *			*1.168 *	*0.930 *	

DAF (%)								
C	42.1 ± 0.9	43.8 ± 0.9	*0.139 *	*2.272 *	45.9 ± 0.9	44.9 ± 0.6	*0.363 *	*0.854 *
H	44.7 ± 0.7	45.4 ± 0.6	*0.499 *	*0.463 *	45.2 ± 0.5	44.1 ± 0.3	*0.32 *	*1.027 *
R	43.7 ± 0.9	45.6 ± 0.8	*0.093 *	*2.953 *	45.9 ± 1.2	45.0 ± 0.9	*0.419 *	*0.67 *

*P *	*0.063 *	*0.213 *			*0.771 *	*0.671 *		
*F *	*2.915 *	*1.586 *			*0.262 *	*0.400 *		

IVDMD								
C	55.8 ± 1.3	52.1 ± 1.2	*0.053 *	*3.948 *	50.8 ± 1.0	51.1 ± 0.7	*0.848 *	*0.037 *
H	54.7 ± 1.4	51.8 ± 1.1	*0.129 *	*2.396 *	51.2 ± 1.0	52.2 ± 0.5	*0.439 *	*0.615 *
R	57.4 ± 1.5	52.3 ± 1.3	*0.080 *	*7.760 *	59.8 ± 1.3	50.0 ± 1.0	*0.546 *	*0.373 *

*P *	*0.331 *	*0.970 *			*0.963 *	*0.266 *		
*F *	*1.123 *	*0.032 *			*0.039 *	*1.366 *		

CP (%)								
C	5.6 ± 0.3	4.9 ± 0.2	*0.255 *	*1.331 *	4.1 ± 0.1	3.8 ± 0.2	*0.254 *	*1.354 *
H	5.5 ± 0.2	4.7 ± 0.5	*0.152 *	*2.13 *	4.0 ± 0.2	3.9 ± 0.2	*0.644 *	*0.218 *
R	6.6 ± 0.7	4.4 ± 0.3	*0.060 *	*6.493 *	3.6 ± 0.2	3.7 ± 0.2	*0.801 *	*0.065 *

*P *	*0.096 *	*0.602 *			*0.143 *	*0.683 *		
*F *	*2.444 *	*0.508 *			*2.051 *	*0.383 *		

*P* and *F* are values of variance analysis followed by a Tukey test.
